# Peptide Inhibitor of Complement C1 (PIC1) Rapidly Inhibits Complement Activation after Intravascular Injection in Rats

**DOI:** 10.1371/journal.pone.0132446

**Published:** 2015-07-21

**Authors:** Julia A. Sharp, Pamela S. Hair, Haree K. Pallera, Parvathi S. Kumar, Clifford T. Mauriello, Julius O. Nyalwidhe, Cody A. Phelps, Dalnam Park, Nicole M. Thielens, Stephen M. Pascal, Waldon Chen, Diane M. Duffy, Frank A. Lattanzio, Kenji M. Cunnion, Neel K. Krishna

**Affiliations:** 1 Department of Microbiology and Molecular Cell Biology, Eastern Virginia Medical School, Norfolk, Virginia, United States of America; 2 Department of Pediatrics, Eastern Virginia Medical School, Norfolk, Virginia, United States of America; 3 The Leroy T. Canoles Jr. Cancer Research Center, Norfolk, Virginia, United States of America; 4 Univ. Grenoble Alpes, IBS, F-38044, Grenoble, France; 5 CNRS, IBS, F-38044, Grenoble, France; 6 CEA, IBS, F-38044, Grenoble, France; 7 Department of Chemistry and Biochemistry, Old Dominion University, Norfolk, Virginia, United States of America; 8 Department of Physiological Sciences, Eastern Virginia Medical School, Norfolk, Virginia, United States of America; 9 Children’s Specialty Group, Norfolk, Virginia, United States of America; University of Leicester, UNITED KINGDOM

## Abstract

The complement system has been increasingly recognized to play a pivotal role in a variety of inflammatory and autoimmune diseases. Consequently, therapeutic modulators of the classical, lectin and alternative pathways of the complement system are currently in pre-clinical and clinical development. Our laboratory has identified a peptide that specifically inhibits the classical and lectin pathways of complement and is referred to as Peptide Inhibitor of Complement C1 (PIC1). In this study, we determined that the lead PIC1 variant demonstrates a salt-dependent binding to C1q, the initiator molecule of the classical pathway. Additionally, this peptide bound to the lectin pathway initiator molecule MBL as well as the ficolins H, M and L, suggesting a common mechanism of PIC1 inhibitory activity occurs via binding to the collagen-like tails of these collectin molecules. We further analyzed the effect of arginine and glutamic acid residue substitution on the complement inhibitory activity of our lead derivative in a hemolytic assay and found that the original sequence demonstrated superior inhibitory activity. To improve upon the solubility of the lead derivative, a pegylated, water soluble variant was developed, structurally characterized and demonstrated to inhibit complement activation in mouse plasma, as well as rat, non-human primate and human serum *in vitro*. After intravenous injection in rats, the pegylated derivative inhibited complement activation in the blood by 90% after 30 seconds, demonstrating extremely rapid function. Additionally, no adverse toxicological effects were observed in limited testing. Together these results show that PIC1 rapidly inhibits classical complement activation *in vitro* and *in vivo* and is functional for a variety of animal species, suggesting its utility in animal models of classical complement-mediated diseases.

## Introduction

The human complement system is an essential component of human inflammatory responses and innate immunity. Complement also plays a central role in adaptive immunity and homeostasis (reviewed in [1]). The complement system consists of three distinct pathways: the classical, lectin and alternative pathways (reviewed in [2]). C1q and mannose binding lectin (MBL) are structurally related initiator molecules of the classical and lectin pathways, respectively. Whereas the ligands for C1q are IgM and clustered IgG, MBL specifically recognizes and is activated by mannan polysaccharide. Activation of C1q and MBL results in the sequential activation of C4 and C2 forming the classical and lectin pathway C3 convertase (C4bC2a). In contrast to the classical and lectin pathways, the alternative pathway does not require a recognition molecule, and is the key pathway for complement cascade amplification. Classical, lectin and alternative pathway activation produces inflammatory mediators (C3a, C5a) and the membrane attack complex (MAC) which results in cellular lysis. Dysregulated control of the complement system is the cause of many autoimmune and inflammatory human diseases [3], including antibody-initiated, complement-mediated disorders such as cold-agglutinin disease [4], acute intravascular hemolytic transfusion reaction [5] and acute/hyperacute transplantation reaction [6], in addition to others.

Our research group has previously demonstrated that the coat protein (CP) of human astrovirus type 1 binds C1q and MBL inhibiting activation of the classical and lectin pathways of complement, respectively, and downstream complement effector function [7, 8]. By binding C1q, CP displaces the serine protease complex (C1s-C1r-C1r-C1s) that is normally bound to the collagen-like tails (CLR) of C1q and thus prevents C1s cleavage [8]. Analogously, CP was also found to bind the region of the CLR of MBL which interacts with the MASP-1 and MASP-2 binding site on this molecule [8]. The C1q/MBL binding region of the astrovirus CP was subsequently narrowed to a 15 amino-acid peptide [9] which underwent extensive modification to yield the 15 amino-acid derivative Polar Assortant (PA): IALILEPICCQERAA [10]. We refer to this family of inhibitory peptides as Peptide Inhibitors of Complement C1 (PIC1) (reviewed in [11]). PA exhibited superior activity as assessed by hemolytic assays with factor B-depleted human serum and inhibition of human erythrocyte lysis in an ABO incompatibility hemolytic assay [10]. Additionally, as proof-of-concept of the potential of this peptide to inhibit complement activation *in vivo*, PA was demonstrated to cross the species barrier and inhibit complement activation in Wistar rats [10]. While PA was able to significantly reduce complement activation *in vivo*, the relative insolubility of the peptide limited further pharmacodynamic testing in rats.

In the current study, we characterize the biochemical properties of the PA peptide and its ability to bind C1q, MBL and ficolins. A fully water soluble pegylated PA peptide was demonstrated to inhibit hemolysis in mouse plasma, as well as rat and non-human primate serum. Preliminary structural characterization of this lead derivative was undertaken and its ability to dose-dependently inhibit complement activation in Wistar rats after intravenous injection is reported.

## Material and Methods

### Ethics statement

Human blood was obtained from healthy volunteers for the generation of serum used in these studies, per the Eastern Virginia Medical School IRB protocol 02-06-EX-0216. Study participants provided written informed consent. Collection of monkey, rat, and mouse blood for the generation of serum or plasma was performed under approved protocols by the Eastern Virginia Medical School Institutional Animal Care and Use Committee and were conducted in accordance with the National Institutes of Health’s Guide for the Care and Use of Laboratory Animals. All procedures were performed under anesthesia and all efforts were made to minimize suffering. While under sedation, rats were kept on a heating pad and monitored every 15 minutes using a pulse oximeter to check heart rate and percent oxygen saturation. Respiration and behavior were visually monitored. Adult cynomolgus macaques (*Macaca fascicularis*) were used for this study. All monkey protocols and procedures were approved by the Institutional Animal Care and Use Committee and were conducted in accordance with the NIH Guide for the Care and Use of Laboratory Animals. Monkeys were maintained at EVMS in a USDA and AAALAC-accredited facility in a 12h light/12h dark environment and fed a diet of standard monkey chow supplemented daily with fresh fruits and vegetables. Monkeys were socially-housed, had access to a variety of manipulatable objects, and received audio/visual stimulation daily. Monkey blood samples were obtained by femoral venipuncture under ketamine chemical restraint (10mg/kg body weight, IM). For this study, the collection of human and animal blood was specifically approved by the EVMS IRB and IACUC, respectively.

### Serum, plasma, purified proteins, peptides, and sheep erythrocytes

Normal human serum (NHS) was prepared as described previously [12]. Non-human primate (NHP) serum was prepared from cynomolgus macaques blood collected as previously described [13] and allowed to clot at room temperature for 1h and then cooled on ice for 2h. Samples were centrifuged and serum was collected as the supernatant. Mouse plasma from four BKS.Cg-Dock7^m+/+Lepr^d8/J control mice (Jackson labs) was generously donated by Dr. Anca Dobrian, EVMS. Mice were euthanized using CO_2_ asphyxia and blood was collected into 1mL heparinized tubes immediately following euthanasia by intracardiac puncture. Plasma was separated by centrifugation at 250×*g*, for 30min at 4°C. Rat plasma was separated from rat blood, collected from intrajugular catheterized male Wistar rats (Harlan) into EDTA-coated tubes, followed by centrifugation at 4°C; the supernatant was saved as plasma. Commercial Wistar rat serum (complement sufficient) was purchased from Innovative Research, Inc. Serum and plasma were stored at -80°C. Lyophilized peptides at ≥90% purity were obtained from New England Peptide (NEP). Purified complement proteins and human factor-B depleted serum were purchased from CompTech. Sheep erythrocytes and anti-sheep erythrocyte-IgM were purchased from MP Biomedicals, LLC and Rockland, Inc., respectively. Antibody-sensitized sheep erythrocytes (EA) were prepared as described previously (7).

### Buffers

GVBS-EDTA (veronal buffered saline with 0.1% gelatin and 0.01M EDTA), GVBS^++^ (veronal buffered saline with 0.1% gelatin, 0.15mM CaCl_2_ and 1mM MgCl_2_).

### C1q binding to immobilized PA

Acetylated PA (AcPA), which behaves identically to PA (7), was adsorbed to wells of Nunc MaxiSorp plates at 2.5μM in 50μL of carbonate buffer (0.06M Na_2_CO_3_, 0.36M NaHCO_3_, pH 9.6) overnight at 4°C. Wells were washed with PBS containing 0.05% Tween (PBST) then blocked with 3% BSA/PBST for 2h at room temperature. Purified C1q (1μg) was added to wells containing 50mM HEPES (pH 7.0) and various concentrations of NaCl and incubated for 1h at room temperature. C1q binding was assessed using a 1:1000 dilution of goat anti-C1q IgG (CompTech) for 1h followed by a 1:1000 dilution of donkey anti-goat HRP IgG (Santa Cruz Biotechnology, Inc.) for 1h, developed with TMB substrate (Thermo Scientific), stopped with 1N H_2_SO_4_, and read at 450nm in a spectrophotometer. Absorbance values from control wells not containing C1q or additional salt were subtracted as background.

### Cross-linking of biotinylated PA to C1q

Purified C1q and biotinylated PA, at a ratio of 1:25, were incubated in conjugation buffer (0.1M Na_2_HPO_4_, 0.15M NaCl, pH 8.0) at room temperature for 2.5h. The crosslinker BS^3^ (Bis(sulfosuccinimidlyl) suberate, Pierce) was added to a final concentration of 20μM and the mixture was incubated for 30 min. The reaction was quenched with 1M Tris-HCl (pH 7.5) to a final concentration of 50μM for 15 min. Reactions were analyzed via SDS-PAGE (NU-PAGE Bis/Tris 4–12% gradient gel) and western blot using a 1:1500 dilution of streptavidin IR 680 (LiCor) for 1h, 1:2000 dilution of goat anti-C1q IgG (CompTech) for 1h and 1:10,000 dilution of donkey anti-goat IgG IR 800 (Licor) for 1h. Gels were analyzed on an Odyssey Infrared Imaging System (Licor) and identical gels were visualized with bio-safe Coomassie stain (Bio-Rad) to confirm total protein content.

### C1q CLR (collagen-like region) generation

Purified C1q in 0.1M NaOAc buffer (pH 4.4), was digested with freshly made porcine pepsin (Sigma) at a ratio of 1:3 for 20h at 37°C. The digestion was stopped by the addition of 1M Tris (pH 9.0) to a final concentration of 45mM. CLR was purified using 100k and 50k MWCO centrifugation filters (Amicon). Samples were assessed by Coomassie-stained 15% SDS-PAGE, under reducing conditions, with purified C1q as a control to confirm CLR presence.

### Surface plasmon resonance (SPR)

AcPA binding to C1q CLR was performed at room temperature using a BI-3000 (Biosensing, USA) with SPR Control 2.4.2. C1q CLR was diluted to 134.4nM in 10mM formic acid (pH 3.0) and approximately 4,200 RU immobilized on a dextran-coated sensor chip (Biosensing, USA) using amine-coupling chemistry in PBS (pH 7.2–7.4). AcPA was diluted to 98, 196, 294 and 392 μM in PBS (pH 7.2–7.4) and binding was measured at a flow rate of 30μl/min in PBS (pH 7.2–7.4). The binding signal was obtained by subtracting background signal generated from an activated then inactivated blank reference channel. Regeneration of the sensor surface was achieved by injection of 10mM NaOH. Data analysis was conducted using SPR Data Analysis 2.4.2 and Kinetic Analysis 2.0.0.3 software (Biosensing, USA) by global fitting into a 1:1 Langmuir binding model. Equilibrium dissociation constant (KD) was calculated from the ratio of dissociation and association rate constants (koff/kon). Data was derived from two or more experiments.

### MBL binding to PA

AcPA and the control peptide CP2 [9] were immobilized to a microtiter plate, as described above, at 25nM. Plates were blocked with 3% BSA/PBST for 2h. Recombinant MBL and K55Q MBL [8,14] were added (5μg/mL in blocking buffer in a total volume of 75μL) and incubated for 1h at room temperature. MBL binding was assessed using a dilution of 1:1000 goat anti-MBL serum (a kind gift from Dr. Neeno Rawal, CompTech) for 1h and a 1:2000 dilution of donkey anti-goat HRP IgG (Santa Cruz Biotechnology, Inc.) for 1h, developed with TMB substrate (Thermo Scientific), stopped with 1N H_2_SO_4_, and read at 450nm in a spectrophotometer. Absorbance values from control wells not incubated with MBL were subtracted as background.

### PA binding to ficolins

Wild-type recombinant ficolins L (ficolin-2), H (ficolin-3), M (ficolin-1) [14, 15] were adsorbed to a microtiter plate at 10μg/mL. C1q (25nM) and BSA (30μg/mL) were immobilized as controls. Plates were blocked with 3% BSA/PBST for 2h. Biotinylated PA was added at various concentrations in blocking buffer containing 0.4M NaCl (to reduce nonspecific binding) and incubated for 1h at room temperature. Following washing, wells were probed neutravidin-HRP (1:4000) in blocking buffer for 1h and developed as described above. Absorbance values from wells not incubated with biotinylated PA were subtracted as background.

### Coat protein (CP) binding to ficolins

The ficolins as described above were adsorbed to a microtiter plate at 2.5μg/mL. C1q and BSA at the same concentration were immobilized as controls. Plates were blocked with 3% BSA/PBST for 2h. Purified astrovirus CP [7] was added at various concentrations in 3% BSA/PBST and incubated for 1h at room temperature. Following washing, wells were probed with a 1:1000 dilution CP antibody [8] in blocking buffer for 1h followed with a 1:4000 dilution of anti-rabbit HRP (Sigma) and developed as described above. The C1q binding was assessed using a 1:1000 dilution of goat anti-C1q IgG (CompTech) for 1h followed by a 1:1000 dilution of donkey anti-goat HRP IgG (Sigma).

### Displacement assay using PA and C1

This was performed similarly to the competitive binding assay described by Hair et al., 2010 [8], with the following differences. Plates were coated with monoclonal antibody to C1q (Quidel) at 1:1000 overnight. In blocking buffer containing 2mM CaC1_2_, partially purified C1 complex was added to wells at a constant amount (0.05μg) with decreasing amounts of either CP or PA, starting with concentrations for CP at 0.145μM and 1.45μM for PA. Detection of C1q and C1s was confirmed as described by Hair et al., 2010 [8].

### Hemolysis assay using factor-B depleted serum

Following the assay described by Mauriello et al., 2013 [10] using factor-B depleted serum and EA, PA derivatives (arginine or glutamic acid substitutions and pegylated versions of PA) were compared to the parent compound PA or AcPA for hemolysis inhibition. Soluble peptides were dissolved in water whereas insoluble peptides were reconstituted in DMSO. Results were standardized to water or DMSO, as appropriate.

### Hemolysis assay using sensitized human AB erythrocytes

Human AB erythrocytes were sensitized as described by Bonaparte et al., 2008 [7] using rat anti-human glycophorin A (Lifespan) IgG. Sensitized cells (5×10^7^), peptide, serum, and GVBS ^++^ were combined in a total volume of 375μL in borosilicate glass tubes and incubated for 1h at 37°C. The reaction was stopped by the addition of 2mL GVBS-EDTA. Tubes were centrifuged and the supernatant was measured in a spectrophotometer at 412 nm to quantitate free hemoglobin. Variations of this assay include no peptide (buffer control or *ex vivo* testing following peptide administration) or assessing the effects of various buffers.

### Non-human primate (NHP) hemolysis assay

Increasing amounts of PA-dPEG24 in 10mM Na_2_HPO_4_ with 0.9% NaCl were incubated with 60μL of pooled NHP serum for 15 min at room temperature. GVBS^++^ and 5×10^7^ sensitized human AB erythrocytes were added to a total volume of 375μL. Samples were placed at 37°C for 1h then stopped with 2mL GVBS-EDTA. Free hemoglobin was quantitated as described above.

### Rat serum hemolysis assay

In a total volume of 375μL, 1x10^9^ human AB erythrocytes (non-sensitized) were combined with various concentrations of PA-dPEG24 (dissolved in 0.1M Na_2_HPO_4_, 0.9% NaCl, pH 7.4) and 50μL of Wistar rat serum (Innovative Research, Inc.) for 15min at 37°C. The reaction was stopped with 2mL GVBS-EDTA and assessed for free hemoglobin, as described above.

### Mouse plasma hemolysis assay

In a total volume of 100μL, 50μL of pooled mouse plasma and various concentrations of PA-dPEG24 dissolved in 0.9% NaCl were combined with 5×10^6^ EA in GVBS^++^ for 30min at 37°C. The assay was stopped with 0.2mL of GVBS-EDTA, and the supernatant was assessed for free hemoglobin, as described above.

### Rat serum hemolysis assay testing of PA-dPEG24 dissolved in various buffers

PA-dPEG24 was dissolved separately in the following buffers: 0.9% NaCl, 0.45% NaCl, 10mM Na_2_HPO_4_ with 0.9% NaCl or deionized H2O. Soluble PA-dPEG24 in individual buffers was then tested in our standard hemolysis assay using sensitized human AB erythrocytes.

### Mass spectrometry

To analyze the oligomeric state of PA-dPEG24, matrix assisted laser desorption ionization time of flight mass spectrometry (MALDI-TOF-MS) was performed on peptide in 0.1% formic acid on an Ultraflex II mass spectrometer (Bruker Daltonic, Billerica, MA) with measurements in both the reflectron and linear mode using α-cyano 4-hydroxycinnamic matrix. The reflectron mode has a higher resolution compared to the linear mode, but only measures effectively up to 4500Da, whereas the linear mode method has a higher mass range with measurements up to 20kDa. Thus, as the predicted mass of PA-dPEG24 is 2773 Da, to detect any larger peptide oligomers, both the reflectron and linear mode were utilized.

### Circular dichroism (CD)

CD spectrum was recorded on a Jasco J-815 CD spectrometer. The peptide concentration was 0.2mg/ mL (72μM) in phosphate buffered saline (8.1mM Na_2_HPO_4_, 137mM NaCl, 1.47mM KH_2_PO_4_, 2.68mM KCl, pH 7.0) in a 1mm path-length cell. The spectrum was recorded at 25°C after a 5 minute equilibration period with a scanning speed of 1 nm per minute, a spectral bandwidth of 5nm and processed with a 10nm smoothing function.

### Nuclear Magnetic Resonance (NMR)

The NMR sample buffer was identical to that described above for CD spectroscopy, with the addition of 10% D_2_O for field lock. The peptide concentration was 1.8mg/mL. A one-dimensional ^1^H NMR spectrum was acquired using 256 transients with 16384 data points per fid and a sweep width of 8012.58 Hz on a Bruker Avance 400 MHz spectrometer at a temperature of 294 K. A W5 watergate sequence [16] was used for solvent suppression. Data was processed and analyzed with ACD/NMR Processor software [17].

### *In vivo* dosing of PA-dPEG24 in rats

PA-dPEG24 was dissolved in 0.9% NaCl at various concentrations (10, 20, or 30mg/mL). Male Wistar rats bearing an intrajugular catheter (Harlan) of approximately 250g each received 1mL of peptide intravenously via the catheter. Control groups received vehicle only or no injection (sham). Blood was drawn from the catheter prior to peptide administration then at various time points post-injection. Plasma from these samples was tested for complement activity in our standard hemolysis assay with sensitized human AB erythrocytes (without peptide) and using 25μL of plasma. In total, 4 doses plus vehicle and sham controls were tested in 4 separate experiments (n = 46) for time points up to 1h. An additional experiment assessing up to 24h post-injection using three doses (10, 20, or 30mg/mL, n = 4 for each group) in 10mM Na_2_HPO_4_, 0.9% NaCl and a vehicle control group (n = 3) was also conducted.

### Statistical analysis

ELISA data were analyzed using a two-tailed, paired, Student's *t* test.

## Results

### PA peptide binds specifically to the CLR of C1q, MBL and ficolins

**Fig 1:**
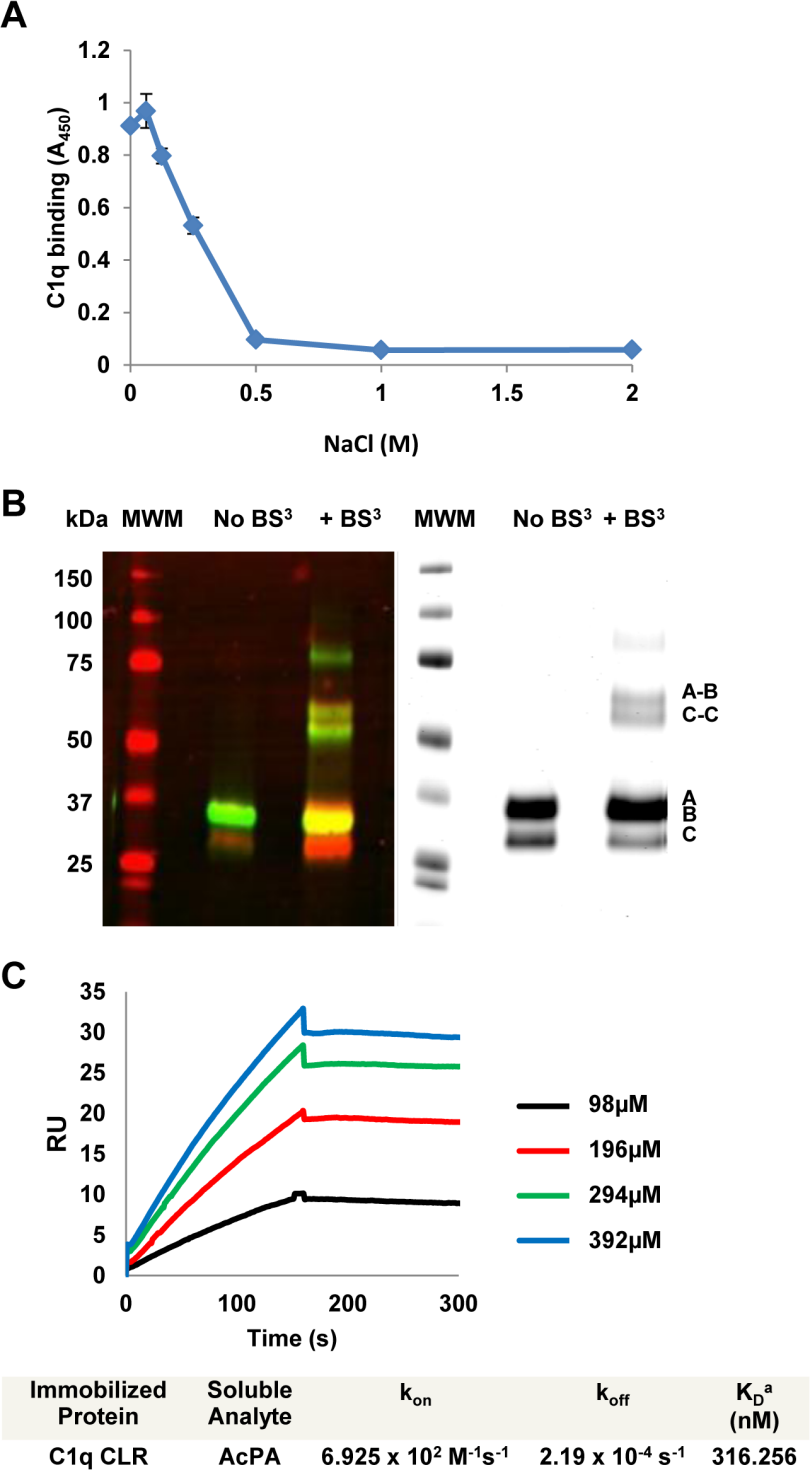
PA interacts with C1q in a salt-sensitive manner and specifically binds the C1q CLR. (A) The AcPA peptide was adsorbed to a microtiter plate and incubated with a constant amount of C1q in the presence of varying amounts of NaCl. Bound C1q was detected with a goat polyclonal anti-C1q sera followed by HRP-conjugated anti-goat sera. Data represent the means of three independent experiments. Error bars denote SEM. (B) C1q and biotinylated PA in the absence or presence of the crosslinker BS^3^ were loaded onto a 4–12% SDS-PAGE gel, (left panel) transferred to nitrocellulose and probed with antibody to detect C1q (green) and streptavidin conjugate to detect PA (red). An identical gel was stained with bio-safe Coomassie blue, and visualized by Odyssey imaging (right panel). Molecular size markers (in kDa) are indicated to the left and chains of C1q (A, B, C, A-B, C-C) are indicated to the right. (C) Interactions between AcPA and the CLR of C1q as measured by surface plasmon resonance (SPR). The CLR of C1q was immobilized onto the surface of a dextran sensor chip and AcPA was injected at the indicated concentrations. Data analysis consisted of globally fitting the SPR sensorgrams for different peptide concentrations with a 1:1 model taking into account mass transport. K_D_ values were calculated from k_off_/k_on_ for each experiment and were averaged from at least two separate experiments.

Previous work from our laboratory identified the 15 amino-acid Polar Assortant (PA) peptide (amino-acid sequence IALILEPICCQERAA) which can bind efficiently to the collagen-like region (CLR) of C1q to inhibit classical pathway complement activation [10]. To evaluate whether charge plays a role in the interaction of C1q with PA, we performed an ELISA in which an acetylated version of the PA peptide was bound to the plate and incubated with C1q in the presence of increasing amounts of sodium chloride. PA and AcPA have identical activity as previously demonstrated [10] and are used interchangeably in the *in vitro* experiments reported here. C1q bound to AcPA in 0mM NaCl and continued to show robust binding through the range of physiological saline (0.154M). However, above 0.25M NaCl, a dose-dependent inhibition of C1q binding to AcPA was observed (p < 0.005), suggesting that the interaction between AcPA and C1q is ionic in nature (Fig 1A). In an attempt to define the region of C1q that is bound by PA, a C-terminally biotinylated PA peptide was incubated with C1q either in the absence or presence of the water-soluble crosslinker BS^3^ (ThermoFisher). BS^3^ contains an amine-reactive N-hydroxysulfosuccinimide (NHS) ester at each end of an 8-carbon spacer arm and reacts with primary amines. In the case of PA, the only primary amine is located at the N terminus of the peptide. The reactions were analyzed by SDS-PAGE followed by immunoblot and Odyssey imaging to detect C1q (green signal) and the biotinylated PA peptide (red signal), respectively. In the samples that were not crosslinked, C1q A and B chains were present in green, indistinguishable as a single band, whereas the C chain is red (Fig 1B, left panel). It is not entirely clear why the C chain is red, but this suggests that the biotinylated PA peptide can remain bound to the monomeric C chain even after boiling, reduction and SDS-PAGE. In the sample that received crosslinker, a prominent yellow band indicated specific interaction of the PA peptide with the A and/or B chain of C1q (Fig 1B, left panel). Due to its small size (1643 Da) PA does not cause a detectable size shift. As with the non-crosslinked sample, the C chain was red. In addition to crosslinking monomeric A/B chains, crosslinks of two higher molecular weight species between 50 and 75 kDa shown as weakly yellow species were detected, which most likely correspond to C1q A-B (69 kDa) and C-C (54 kDa) dimers, as previously reported (Fig 1B, left panel) [18]. This confirms that BS^3^ efficiently crosslinks C1q and that biotinylated PA can bind monomeric chains of C1q along with the dimers A-B and C-C. This data is consistent with previous findings from our laboratory showing that astrovirus CP binds to the A/B chain of C1q as assessed by overlay blot analysis [7]. An identical gel stained with bio-safe Coomassie blue was also performed to visualize total proteins (Fig 1B, right panel).

Biotinylated PA was previously shown to specifically bind to the CLR of C1q by ELISA [10]. To evaluate the kinetics of AcPA interaction with the CLR, we utilized surface plasmon resonance (SPR) to measure the affinity of immobilized CLR with AcPA. SPR sensorgrams were generated by injecting different concentrations of AcPA and were fitted to the 1:1 model with mass transport (Fig 1C). The mean apparent equilibrium dissociation constant (K_D_) was 316nM demonstrating that AcPA binds the CLR of C1q with high affinity. Previous work from our laboratory demonstrated that PA bound intact C1q with a K_D_ value of 33.3nM [10]. The difference in K_D_ value between intact C1q and CLR alone most likely reflects changes in the quarternary structure of CLR after removal of the globular head domains or the contribution of interactions of the globular heads of C1q with the PA peptide, or both.

**Fig 2:**
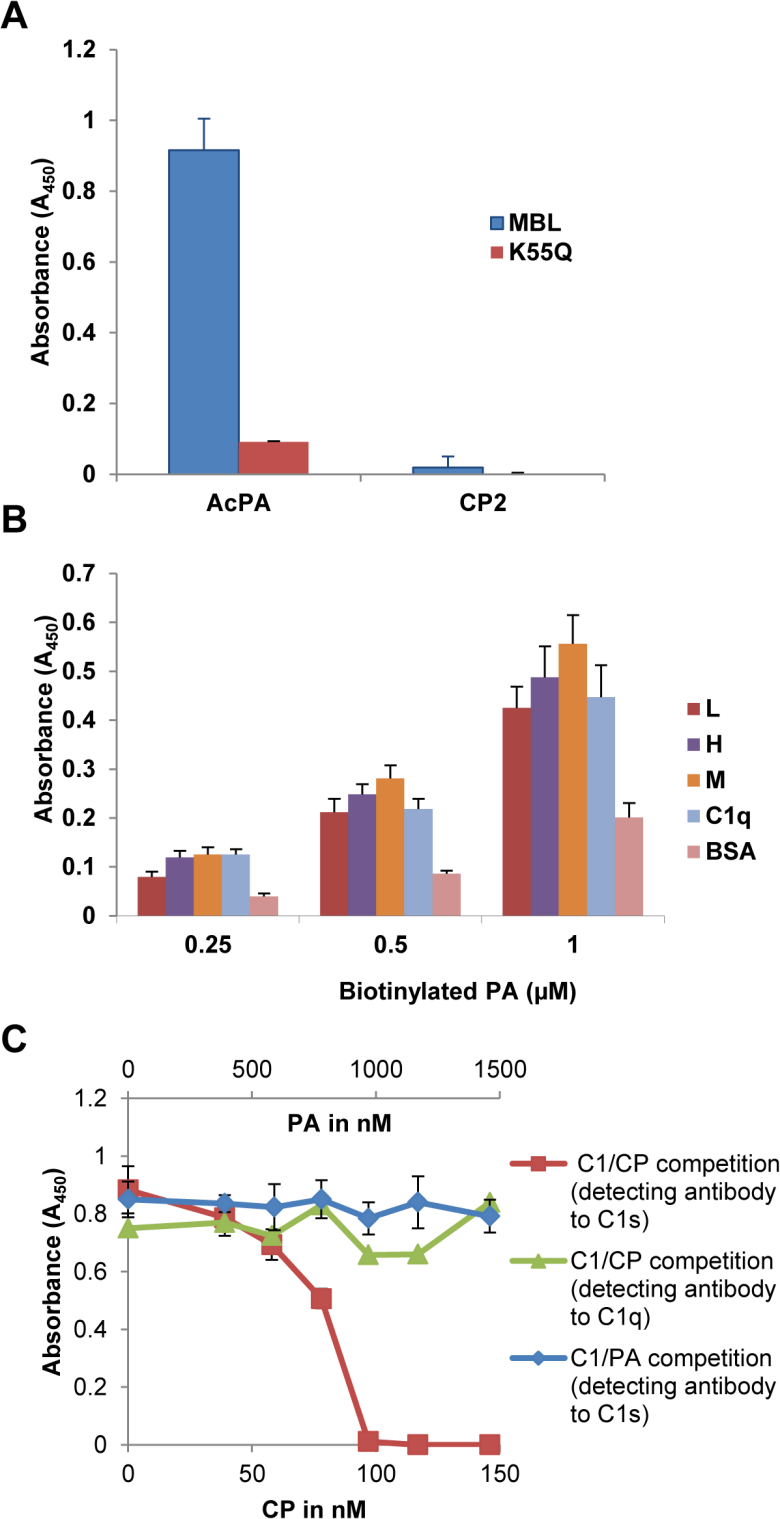
PA binds to MBL and ficolins and does not displace C1s from C1q. (A) The AcPA and CP2 peptides were adsorbed to a microtiter plate and incubated with a constant amount of MBL or K55Q. Bound MBL was detected with a polyclonal goat anti-MBL sera followed by HRP-conjugated anti-goat sera. (B) The various ficolins indicated in the figure were adsorbed to the microtiter plate and incubated with biotinylated PA followed by a neutravidin conjugate. BSA was used as a negative control for binding. (C) Partially purified C1 was mixed with increasing amounts of CP or PA and added to a microtiter plate coated with monoclonal antibody to C1q. After washing, C1s signal present in intact C1 complexes was measured by ELISA using polyclonal antibody to C1s (CP, red line; PA, blue line) or polyclonal antibody to C1q (green line) to confirm C1q was bound to the plate. Data represent the means of three independent experiments. Error bars denote SEM.

Earlier work from our laboratory has demonstrated that the astrovirus coat protein (CP) specifically binds to mannose binding lectin (MBL) and inhibit MBL-dependent activation of the lectin pathway of complement [8]. Interestingly, while CP bound specifically to both purified and recombinant MBL, it did not bind to the MBL variant K55Q [8]. This lysine residue resides in the CLR of MBL and is critical for interaction of MASP-1 and MASP-2 with MBL [14]. To see if AcPA retained the same behavior as CP, AcPA and a negative control peptide (CP2, [9]) were immobilized to a microtiter plate and incubated with either wild-type recombinant MBL or the derivative K55Q. Wild-type MBL efficiently bound to AcPA; however, as with the full-length CP molecule, AcPA did not bind K55Q (Fig 2A). As expected, CP2 was not bound by either form of MBL (Fig 2A). These results demonstrated that analogously to C1q, PA binding to MBL is dependent on residue K55, strongly suggesting that PA binds the CLR region of MBL critical for interaction with its cognate serine proteases.

As PA efficiently bound to MBL, we next tested the binding of this peptide to recombinant human ficolins L, H and M. Ficolins are structurally similar to MBL and C1q being composed of a CLR with a lectin-like recognition domain comprised of three fibrinogen-like domains that bind to acetylated compounds in the context of pathogen- and apoptotic cell-associated molecular patterns [19]. As with MBL, ficolins interact with MASP-1 and MASP-2 via a reactive lysine residue and can thus initiate complement activation via the lectin pathway [20]. Given the structural and functional relatedness to MBL and C1q, we tested whether wild-type L, H and M ficolins could interact with PA. The ficolins were adsorbed to a microtiter plate and then incubated with biotinylated PA. All three ficolins bound PA to the same degree as C1q at various concentrations of biotinylated PA (Fig 2B). Wild-type CP was also demonstrated to bind to these ficolins in a similar manner to C1q but not BSA (S1 Fig).

Previous work in our laboratory demonstrated that the astrovirus CP physically dissociates the C1s-C1r-C1r-C1s tetramer from the C1q CLR, thus inhibiting activation of the C1 complex [8]. To determine if PA was capable of displacing the tetramer in an analogous fashion, we performed a competition ELISA as previously reported [8]. Monoclonal antibody to C1q was adsorbed to the microtiter plate and partially purified C1 complex with increasing amounts of either wild-type CP or PA were added simultaneously. Adherent C1s retained in the C1 complex was detected with a polyclonal antibody to C1s. C1s signal decreased with increasing amounts of CP demonstrating that CP dose-dependently displaces C1s, as previously reported (Fig 2C, red line) [8]. To verify that C1q was firmly attached to the plate in the presence of increasing amounts of CP, bound C1q was confirmed with a polyclonal antibody to C1q (Fig 2C, green line). In contrast to CP, PA did not demonstrate competitive displacement of C1s from C1q even at 10-fold molar excess compared to CP (Fig 2C blue line). Thus, while PA shares many of the functional properties of the parent CP molecule in terms of binding the CLR and inhibiting C1 activation, PA is not able to grossly displace C1s (see Discussion).

### Amino-acid substitution analysis of PA

In order to evaluate which residues of the PA peptide are critical for inhibitory function of the peptide, we performed an arginine and glutamic acid scan of PA to see if substitution of positively or negatively charged residues affects the inhibitory activity of PA in a hemolytic assay using factor B-depleted serum and antibody-sensitized sheep erythrocytes. Substitution of arginine or glutamic acid at any of these positions in PA reduced the ability of PA to inhibit hemolysis, usually with nearly complete loss of function (Table 1). Together with our previous results from an alanine scan [10], these results suggest that the function of PA cannot be easily improved by simple amino-acid substitution.

**Table 1:** Hemolytic assay in factor B-depleted serum of arginine and glutamic acid substitution peptides.

Peptide name and controls	Peptide sequence	Hemolysis (%)
Water	-	100.00
GVBS^++^	-	2.75
DMSO	-	96.32
**PA**	**IALILEPICCQERAA**	**39.49**
PA-I1R	RALILEPICCQERAA	91.93
PA-A2R	IRLILEPICCQERAA	87.28
PA-L3R	IARILEPICCQERAA	80.45
PA-I4R	IALRLEPICCQERAA	43.71
PA-L5R	IALIREPICCQERAA	93.40
PA-E6R	IALILRPICCQERAA	87.59
PA-P7R	IALILERICCQERAA	89.85
PA-I8R	IALILEPRCCQERAA	62.32
PA-C9R	IALILEPIRCQERAA	89.54
PA-C10R	IALILEPICRQERAA	89.80
PA-Q11R	IALILEPICCRERAA	90.87
PA-E12R	IALILEPICCQRRAA	95.83
PA-A14R	IALILEPICCQERRA	88.65
PA-A15R	IALILEPICCQERAR	44.24
PA-I1E	EALILEPICCQERAA	89.27
PA-A2E	IELILEPICCQERAA	92.24
PA-L3E	IAEILEPICCQERAA	100.27
PA-I4E	IALELEPICCQERAA	92.07
PA-L5E	IALIEEPICCQERAA	94.33
PA-P7E	IALILEEICCQERAA	96.10
PA-I8E	IALILEPECCQERAA	95.74
PA-C9E	IALILEPIECQERAA	91.62
PA-C10E	IALILEPICEQERAA	95.21
PA-Q11E	IALILEPICCEERAA	65.60
PA-R13E	IALILEPICCQEEAA	101.99
PA-A14E	IALILEPICCQEREA	98.09
PA-A15E	IALILEPICCQERAE	93.13

The final concentration of peptide in factor B-depleted serum was 0.77mM. Each peptide was evaluated in triplicate and the mean values are reported.

### Optimization of a water-soluble PA derivative

**Fig 3:**
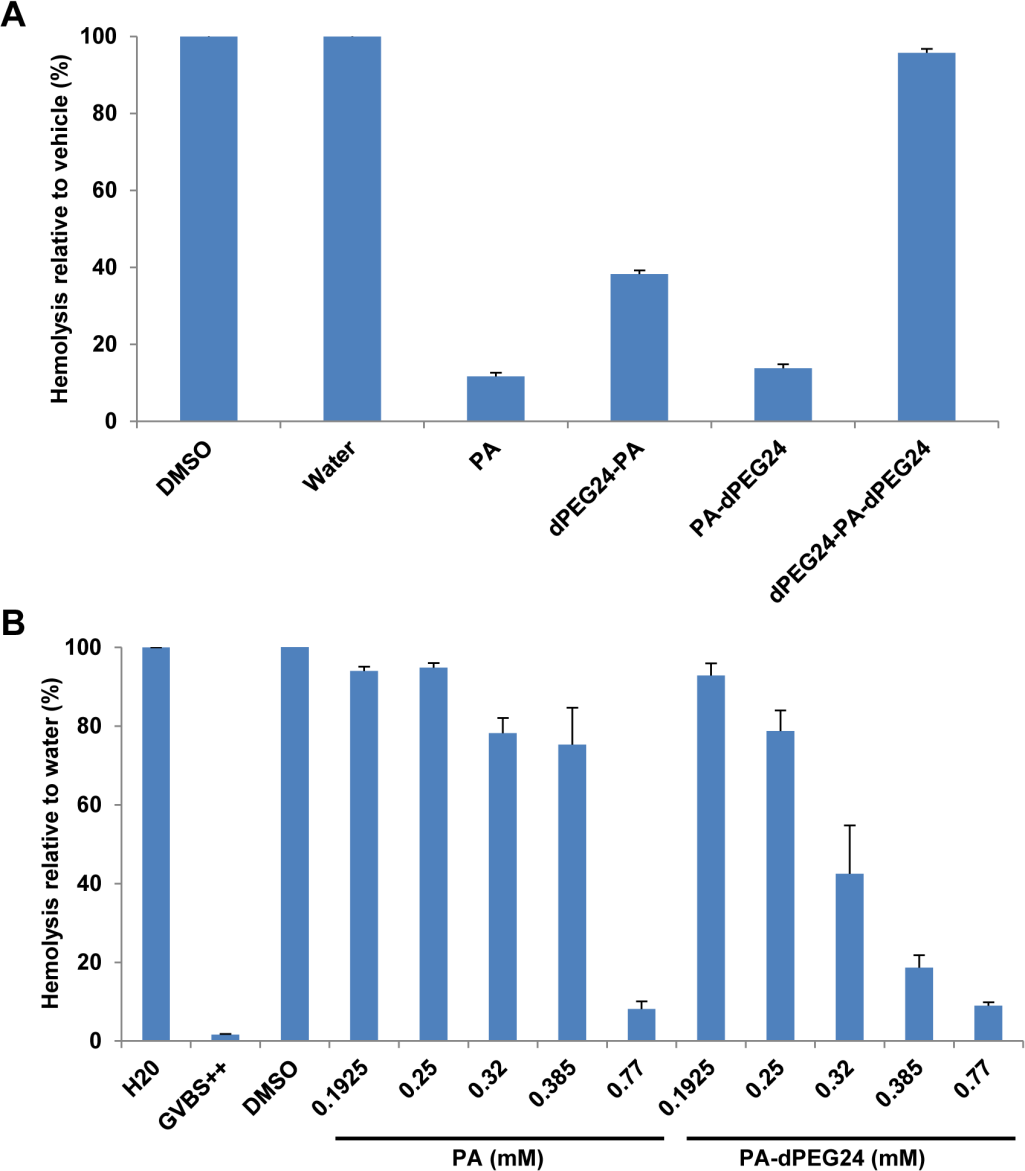
Pegylated versions of PA inhibit complement activity in a hemolytic assay. (A) Hemolytic assays using factor B-depleted serum were performed with PA dissolved in DMSO and its pegylated derivatives dissolved in water. Factor B-depleted serum was incubated with 0.77mM of each peptide and then added to sensitized sheep erythrocytes. (B) Titration of increasing amounts of PA and PA-dPEG24 in the hemolytic assay. Water and DMSO were used as vehicle controls in the presence of factor B-depleted serum. GVBS^++^ is a buffer- only control. Values are the means of three independent experiments. Error bars represent the SEM.

In an attempt to improve the solubility of this peptide, PA was synthesized with a PEG linker consisting of 24 PEG units on either the N terminus (dPEG24-PA), C terminus (PA-dPEG24) or both the N and C termini of PA (dPEG24-PA-dPEG24). All three peptide derivatives were soluble in water. The peptides were then tested for inhibition of complement activity in a hemolytic assay using factor B-depleted serum and antibody-sensitized sheep erythrocytes. PA-dPEG24 in water inhibited complement activation to the same level as PA in DMSO (Fig 3A). dPEG24-PA also inhibited hemolysis, although not to the same degree as PA, whereas dPEG24-PA-dPEG24 completely lost inhibitory activity in the hemolytic assay (Fig 3A). To more carefully evaluate the inhibitory activity of PA-dPEG24 compared to PA, increasing concentrations of both peptides were tested in the hemolytic assay. While both peptides inhibited hemolysis to the same level at high concentrations (0.77mM), PA-dPEG24 demonstrated a gradual dose-dependent response compared to PA (Fig 3B). The difference in titration between PA and the PA-dPEG24 derivative is most likely due to the increased solubility of the pegylated version in an aqueous solution. Additional PA derivatives with decreasing number of C-terminal PEG moieties were synthesized and tested for inhibitory activity in the hemolytic assay. As few as 2 PEG moieties on the C terminus of PA was found to endow PA with solubility in water; however, each of these peptides showed some loss of inhibitory activity in the hemolytic assay (S1 Table).

### Structural characterization of PA-dPEG24

**Fig 4:**
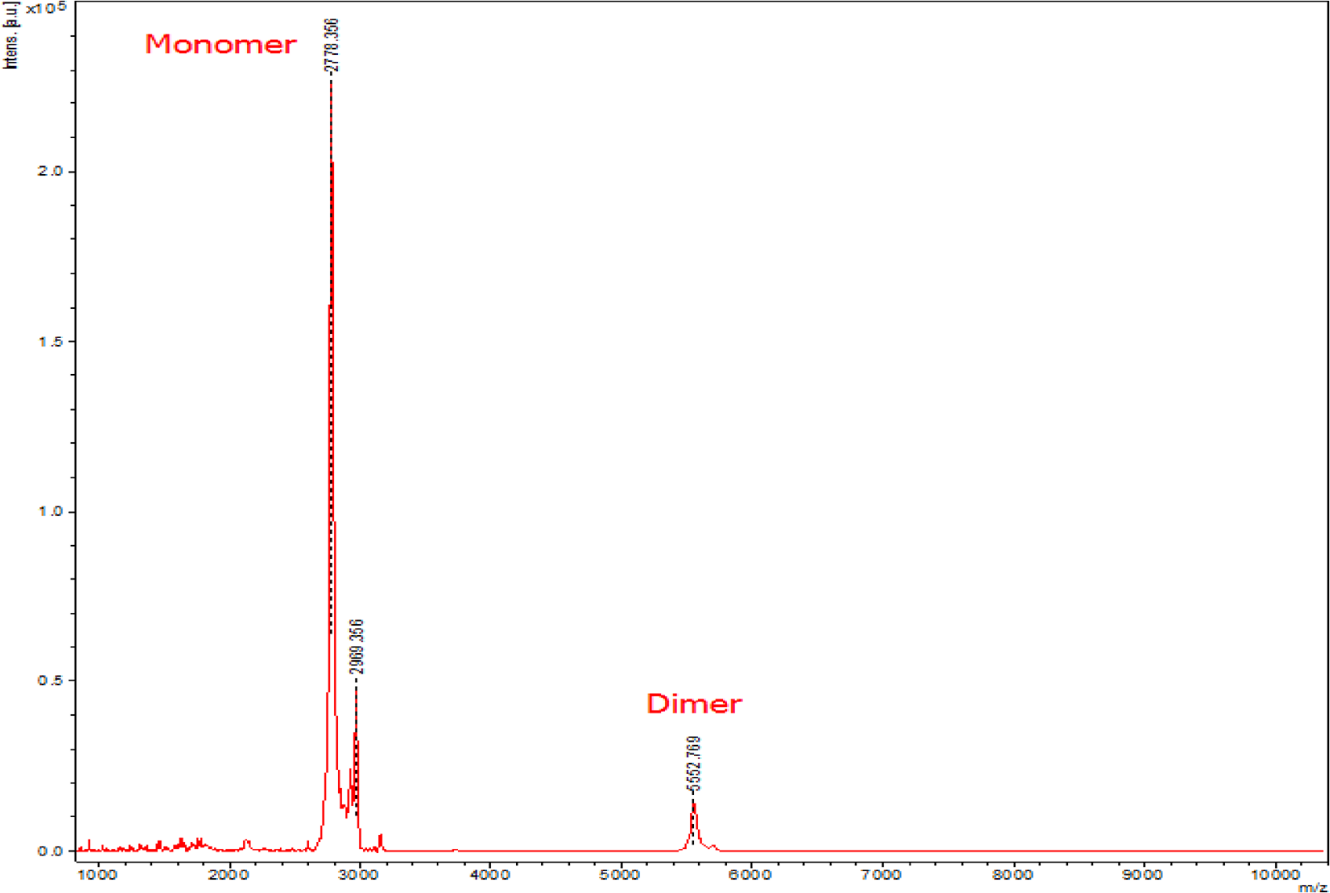
Linear mode MALDI-TOF mass spectrometry analysis of PA-dPEG24 oligomeric state. PA-dPEG24 in acidified water was analyzed by linear mode mass spectrometry. The spectrum shows two peaks for PA-dPEG24 with a monomer peak at 2778 and a dimer form at 5553.

The PA peptide contains two cysteine residues at positions 9 and 10. Given that either cysteine could potentially participate in intermolecular disulphide bonding between two or more peptide molecules, we utilized mass spectrometry to analyze PA-dPEG24 for oligomerization. Matrix assisted laser desorption ionization mass spectrometry (MALDI-TOF MS) was performed with measurements in both the reflectron and linear mode. Linear mode MALDI-TOF mass spectrometry demonstrated that the majority of the peptide formed a peak at 2778 Da which is consistent with a monomer of PA-dPEG24 that has a theoretical mass of 2773 Da (Fig 4). A minor peak at 5553 Da was consistent with a dimerized version of this peptide; however, no other higher molecular weight forms of the peptide were evident (Fig 4). This data suggests that while there may be some dimer formation, the majority of PA-dPEG24 exists as a monomer.

**Fig 5:**
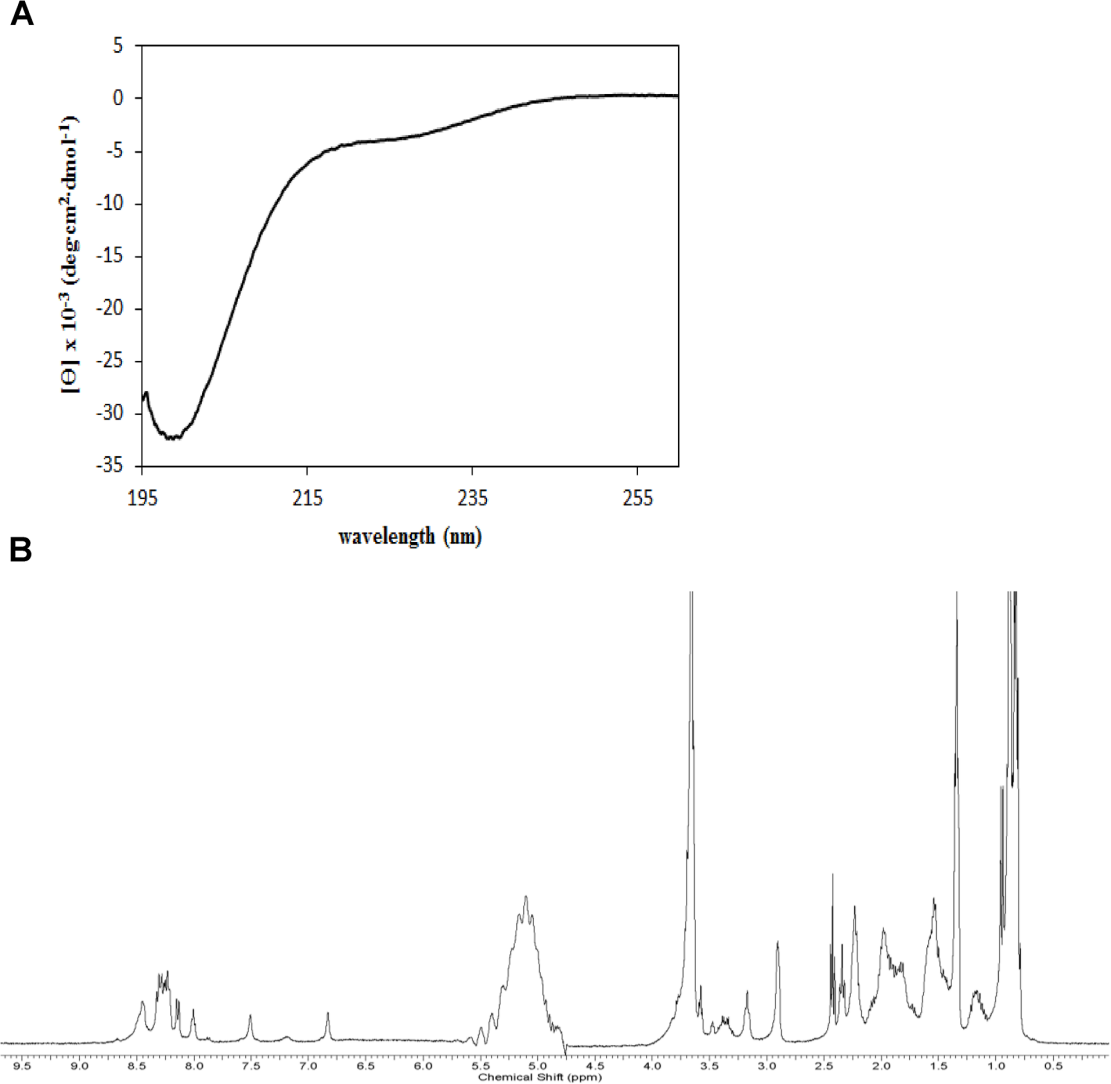
CD and 1D ^1^H NMR of PA-dPEG24. (A) The PA-dPEG24 peptide at 0.2mg/mL in PBS was analyzed on a Jasco J-815 CD spectrometer. (B) The PA-dPEG24 peptide at 1.8mg/mL in PBS with 10% D_2_O was analyzed on a Bruker Avance 400 MHz spectrometer at a temperature of 294 K.

In an attempt to gain more structural information on PA-dPEG24, we performed circular dichroism (CD) and 1 dimensional ^1^H nuclear magnetic resonance (NMR) on this peptide. Based on amino-acid sequence alone, the program PSIPRED [21] predicts a short beta strand at the N terminus and alpha helix in the C-terminal half of the peptide. The CD spectrum of PA-dPEG24 is shown in Fig 5A. Overall, this spectrum is consistent with a largely disordered peptide. The deep negative band near 200nm is normally associated with a lack of regular secondary structure. However, the negative readings in the relatively flat region near 220 to 225nm is suggestive of some alpha helical or beta sheet character. The analysis program CDSSTR [22] fits the observed CD spectrum by predicting 40% alpha helix and 25% beta sheet, though in order to fit, nearly half of these secondary structural elements are predicted as distorted. Thus, this amount of secondary structure may be an overestimation. In summary, the CD spectrum is consistent both with some disorder and some regular secondary structure.

The 1D ^1^H NMR spectrum of the PA-dPEG24 peptide is presented in Fig 5B. The lack of amide peaks outside of the 8.0 to 8.5ppm range and methyl group peaks below 0.5ppm is normally associated with disorder. However, for a small peptide with no aromatic residues, the conclusion is less certain, as the observed chemical shift range for this sort of peptide is expected to be smaller. The primary conclusion is similar to that for CD: the spectrum is consistent with a largely disordered peptide that may contain some alpha helix, and perhaps even some sheet-like structure.

### PA-dPEG24 is active in rat serum, mouse plasma and non-human primate serum

**Fig 6:**
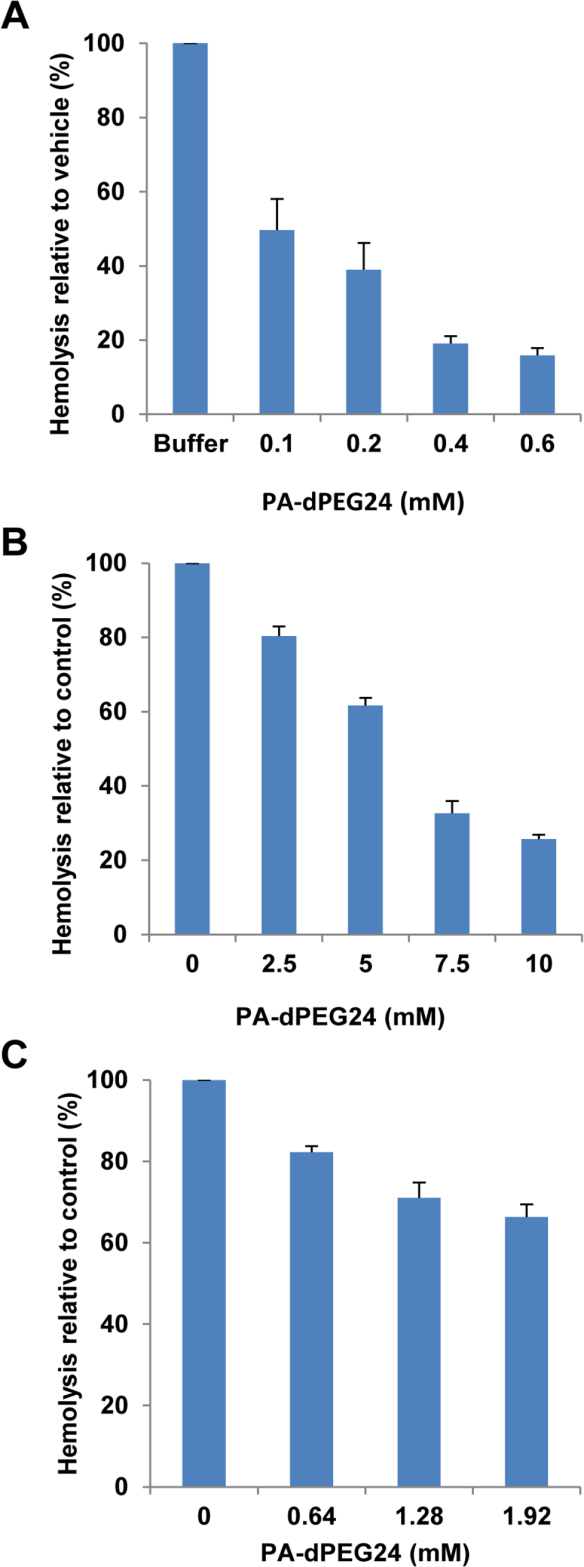
PA-dPEG24 inhibits complement activation in rat serum, mouse plasma and non-human primate serum. Hemolytic assays using (A) Wistar rat serum, (B) mouse plasma and (C) cynomolgus monkey serum were performed with increasing amounts PA-dPEG24 dissolved in 100mM Na_2_HPO_4_ with 0.9% NaCl buffer for rat serum, 10mM Na_2_HPO_4_ with 0.9% NaCl for cynomolgus monkey serum and 0.9% NaCl for mouse plasma. Serum or plasma were incubated with peptide and then added to human AB erythrocytes. Values are the means of three independent experiments. Error bars represent the SEM.

While PA-dPEG24 was able to inhibit complement activity in factor B-depleted human serum, we also wanted to test its activity in serum isolated from rats, mice and non-human primates as a pre-requisite for *in vivo* animal model testing. As previously demonstrated for PA [10], increasing concentrations of PA-dPEG24 were incubated with Wistar rat serum and then added to human AB erythrocytes. We have shown that human AB erythrocyte hemolysis in Wistar rat serum is a classical complement pathway event that models ABO incompatibility *in vitro* [23]. PA-dPEG24 was able to efficiently inhibit complement-mediated lysis from Wistar rat serum in a dose-dependent fashion (Fig 6A). Next, we tested the ability of this peptide to inhibit complement activation in mouse plasma, and showed inhibition of complement-mediated hemolysis in a dose-dependent manner (Fig 6B). To assess if PA-dPEG24 was able to block complement mediated hemolysis in primate serum, we isolated serum from the blood of the cynomolgus monkey (*Macaca fascicularis*) and developed a hemolytic assay using human AB erythrocytes. PA-dPEG24 was able to dose-dependently inhibit complement-mediated lysis by primate serum (Fig 6C). The ability of PA-dPEG24 to specifically inhibit complement activity from the serum or plasma of three different animal species as well as humans demonstrates that PIC1 peptides have broad cross-species functionality.

### *In vivo* inhibition of complement activity in rats by PA-dPEG24

**Fig 7:**
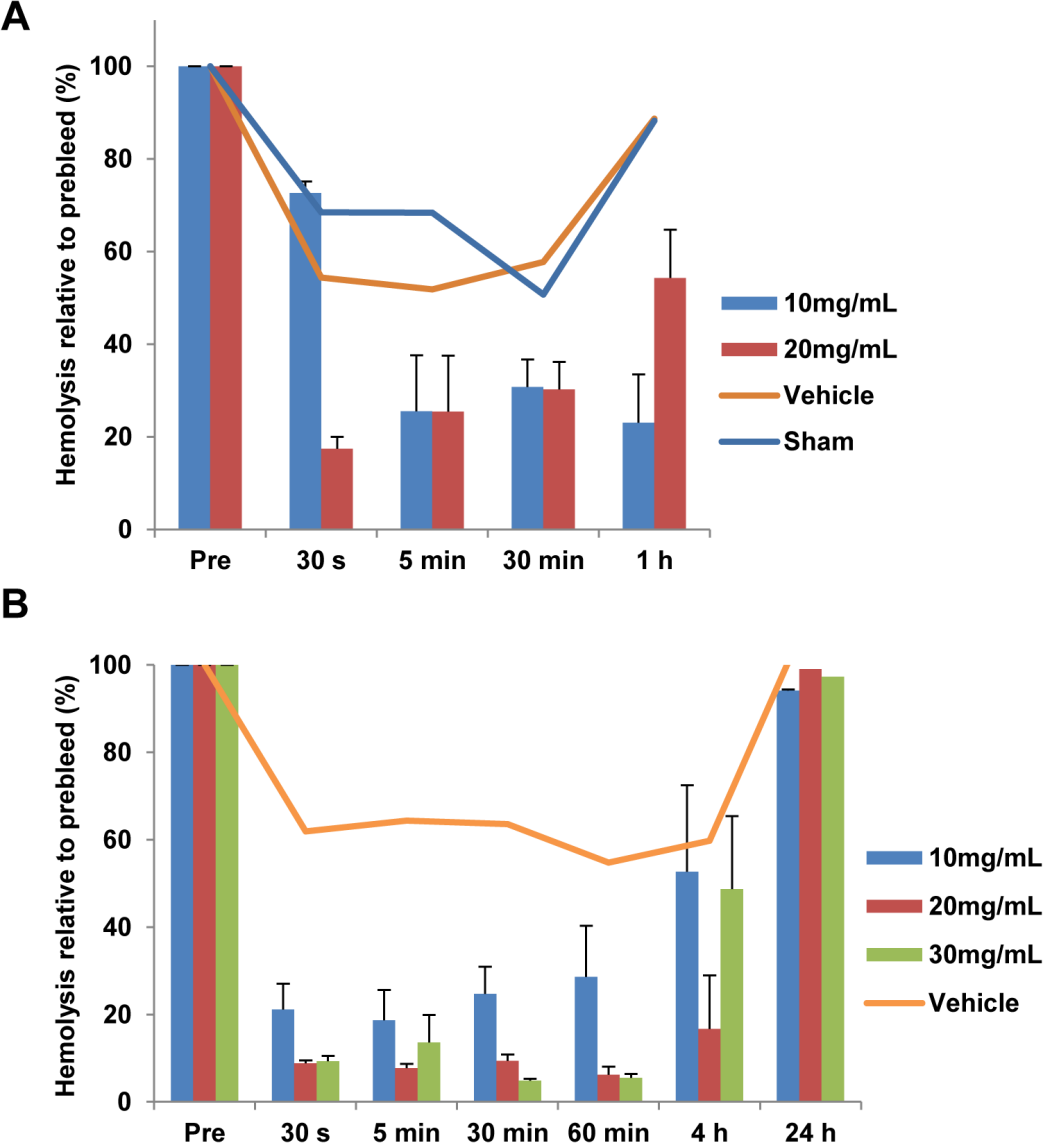
PA-dPEG24 inhibits complement activation *in vivo*. (A) Rats were IV administered PA-dPEG24 at 10 (n = 2) or 20mg/mL (n = 2) in 0.9% NaCl or received a vehicle control (n = 2) or were sham treated (n = 2). B, Rats were IV administered PA-dPEG24 at 10mg/mL (n = 4), 20mg/mL (n = 4) or 30mg/mL (n = 4) in 10mM Na_2_HPO_4_, 0.9% NaCl or received a vehicle control (n = 3). At the indicated time points post-administration, blood was collected and plasma isolated. Plasma was tested in hemolytic assays with human AB erythrocytes. Error bars represent the SEM.

Previous work from our laboratory demonstrated that the PA peptide and an earlier 30 residue PIC1 derivative (E23A) were able to inhibit complement activation in Wistar rats when delivered IV and IP, respectively [10]. However, a serious limitation to these peptide analogs was their lack of solubility in aqueous solution. Given the aqueous solubility of PA-dPEG24, we set out to determine their efficacy *in vivo*. As a prerequisite to testing in rats, a hemolytic assay of PA-dPEG24 dissolved in water or physiological buffers demonstrated that this peptide was able to inhibit complement activity in Wistar rat serum *in vitro* (S2 Fig). To evaluate if PA-dPEG24 delivered intravenously could block complement activation in blood, we performed a dosing study in which this peptide was delivered IV into jugular catheterized Wistar rats (Harlan). Blood was then drawn from the animals at various times post-peptide administration, plasma isolated and a hemolytic assay performed using human AB erythrocytes. In total, 4 doses of 5, 10, 20 and 30mg/mL were tested in rats along with vehicle and sham controls (n = 46) in 4 separate experiments. For simplicity, only two representative experiments are shown. Rats receiving either a 10 or 20mg/mL dose of peptide showed an inhibition of complement activity in a hemolytic assay from plasma purified from blood collected over a 1-hour period (Fig 7A). A separate experiment was performed in which 10, 20 or 30mg/mL of PA-dPEG24 was administered to the rats and complement activity assessed up to 24 hour post-compound administration. All three doses rapidly inhibited complement activation by 30 seconds after administration, with the 20 and 30mg/mL doses inhibiting activation by 90% (Fig 7B). This suppression was maintained for 1 hour with complement activity levels increasing to 50% by 4 hours and completely recovering by 24 hours in all three doses. For both experiments, the vehicle animals as well as sham showed some mild decrease in hemolysis after drawing the preinfusion sample (Fig 7A and 7B). Complete blood counts and blood chemistries were taken at 48 hours for a subset of animals receiving 10mg/mL of compound. No significant differences were seen between the vehicle control animals and animals receiving peptide (S2 Table).

## Discussion

In this study, we explored the structural and biophysical properties as well as *in vitro* and *in vivo* activity of the PIC1 PA derivatives. PA, a 15 amino-acid peptide, was initially derived from the 787 amino acid CP of human astrovirus type 1 [7]. We had previously demonstrated that the CP molecule could specifically bind the A/B chain of C1q to inhibit classical pathway complement activation [7]. The binding of the C1s-C1r-C1r-C1s tetramer to the CLR of C1q is essentially mediated by the reactive, non-hydroxylated lysine residues K61 (B chain) and K58 (C chain) that reside in the CLR of C1q [24]. CP inhibited activation of the C1 complex by displacing the C1s-C1r-C1r-C1s tetramer from the CLR of C1q [9]. Like C1q, MBL is a structurally and functionally similar molecule that harbors a reactive lysine residue (K55). This residue resides in the MBL CLR and is essential for binding the serine proteases MASP-1 and MASP-2 [14], which is equivalent to C1s-C1r-C1r-C1s of the classical pathway [25]. CP was found to inhibit lectin pathway activation and bound MBL, but not the K55Q MBL mutant [8]. Here we demonstrate that PA retains much of the same properties as the parent CP molecule in that it can bind the A/B chain of the CLR of C1q to inhibit classical pathway activation (Fig 1B and 1C) and binds wild-type MBL, but not the K55Q mutant (Fig 2A). We also found that PA and CP bound to the L, H and M ficolins which are structurally and functionally similar to C1q and MBL (Fig 2B and S1 Fig, respectively).

**Fig 8:**
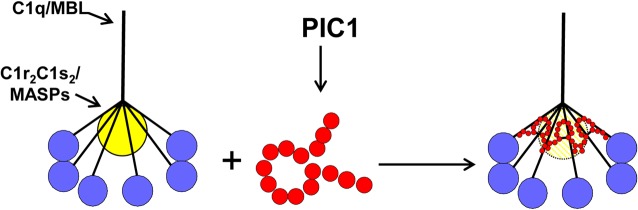
Model of PA inhibition of C1/MBL function. Our data favors a model in which PIC1 derivatives functionally disrupt the C1s-C1r-C1r-C1s/MASP interaction with the CLR of C1q/MBL, respectively. By PIC1 binding to the serine protease interaction site on CLR, the serine proteases are hypothesized to be in a suboptimal conformation for autoactivation and initiation of the classical and/or lectin pathways of complement.

Whereas PA shared the same properties as CP in the assays discussed above, the C1 competition ELISA demonstrated that while CP physically displaced C1s (and thus by extension C1s-C1r-C1r-C1s) from C1q, this was not the case for PA (Fig 2C). We attribute this to the fact that the CP molecule is 787 residues and in the form of a trimer [7] and thus its physical size could feasibly displace the serine protease tetramer. PA in contrast is only 15 residues, but binds to the C1q CLR with a K_D_ value of 316nM and to intact C1q with a K_D_ value of 33nM as assessed by SPR (Fig 1C and Mauriello et al., 2013 [10], respectively). Given that the affinity of C1s-C1r-C1r-C1s for C1q by SPR has been assessed by two independent laboratories as 13nM [24] and 2.72nM [25], it is not unreasonable to suggest that PA, perhaps multiple molecules, can compete with C1s-C1r-C1r-C1s for some of its 6 binding sites on C1q. While not physically displacing C1s-C1r-C1r-C1s, it may be that binding of PA to the CLR is sufficient to disrupt the orientation of the serine protease tetramer preventing autocatalytic cleavage of C1r and activation of the classical pathway. A hypothetical model of the mechanism by which PIC1 derivatives inhibit C1 and MBL activation is presented in Fig 8.

One significant drawback to the parent PA peptide is its lack of solubility in aqueous solutions. Pegylation of PA at the C terminus resulted in the peptide’s ability to be soluble in water or saline solution while retaining inhibitory activity on the classical pathway of complement (Fig 3). The discovery of soluble versions of PA allowed us to further analyze both the structure and function of this peptide. Using the PA-dPEG24 peptide, we determined that this molecule was predominantly monomeric, as assessed by mass spectrometry (Fig 4). The small amount of dimer formed was most likely due to intermolecular disulphide bonding as PA contains cysteines at positions 9 and 10. Structural analysis of PA-dPEG24 by CD and 1D ^1^H NMR demonstrated little ordered structure (Fig 5). Experiments to define the precise region of the C1q CLR that is bound by the peptide along with structural details are currently under investigation in our laboratory.

Given its favorable solubility profile, PA-dPEG24 was tested in hemolytic assays of complement suppression using rat, mouse and non-human primate serum or plasma in preparation for *in vivo* testing. This peptide was able to inhibit complement activation in all three hemolytic assays demonstrating a broad spectrum of inhibitory activity (Fig 6). Dosing of this peptide in Wistar rats showed that a 20mg/mL dose could inhibit complement activation in the animal by >90% in 30 seconds with a functional half-life of 4 hours (Fig 7). Pegylation has been demonstrated to increase the *in vivo* residence of peptidic drugs possibly by preventing their rapid degradation by host proteases [26]. Additionally, no overt toxicity was seen in animals up to 48 hours after compound administration (S2 Table). More detailed pharmacokinetic, pharmacodynamic and toxicity testing of this derivative is presently underway.

There is currently significant interest in the development of complement therapeutics for an array of disease processes in which dysregulated complement plays a role [27]. The classical pathway of complement is associated with a number of antibody-initiated, inflammatory disease processes such as some forms of ischemia-reperfusion injury, hyperacute transplantation rejection, cold agglutinin disease and acute intravascular hemolytic transfusion reaction, among others [4–6, 28]. We have recently developed a rat model of acute intravascular hemolytic transfusion reaction [23] and are in the process of evaluating PIC1 derivatives in this model of antibody-initiated classical pathway disease.

## Supporting Information

S1 Table Solubility and hemolytic assay in factor B depleted serum of PEGylated PA peptides. The final concentration of peptide in factor B-depleted serum was 0.77mM. ^1^Peptides not soluble in water were resuspended in DMSO. ^2^In the hemolytic assay, soluble peptides are standardized to water and insoluble peptides standardized to DMSO.
(DOCX)

S2 Table PA-dPEG24 toxicology evaluation at 48 hr (ranges). (DOCX)

S1 Fig Astrovirus CP binds to ficolins H, M and L. The various ficolins indicated in the figure were adsorbed to the microtiter plate and incubated with CP followed by antibody to CP. BSA was used as a negative control for binding and C1q as a positive control for binding. Ficolin data represent the means of three independent experiments with error bars denote SEM. BSA and C1q values were from two independent experiments.
(TIF)

S2 Fig PA-dPEG24 inhibits complement activation *in vitro* in physiological buffers. Hemolytic assays using Wistar rat serum were performed with PA-dPEG24 dissolved in 0.9% NaCl, 0.45% NaCl, 10 mM Na_2_HPO_4_ with 0.9% saline or water. The sera were incubated with peptide and then added to human AB erythrocytes.
(TIF)
