# Supplementary material for: Peptide Inhibitor of Complement C1 (PIC1) Rapidly Inhibits Complement Activation after Intravascular Injection in Rats
Source: PLoS One. 2015 Jul 21;10(7):e0132446. doi: 10.1371/journal.pone.0132446 (PMC4511006; doi:10.1371/journal.pone.0132446)
Supplement: S1 Table — The final concentration of peptide in factor B-depleted serum was 0.77mM. 1Peptides not soluble in water were resuspended in DMSO. 2In the hemolytic assay, soluble peptides are standardized to water and insoluble peptides standardized to DMSO. (DOCX) [file pone.0132446.s001.docx]

**S1 Table. Solubility and hemolytic assay in factor B depleted serum of PEGylated PA peptides.**

| **Peptide name and controls** | **Peptide sequence** | **Solubility in water^1^** | **Hemolysis (%)^2^** |
| --- | --- | --- | --- |
| Water | - | - | 100.00 |
| DMSO | - | - | 95.17 |
| **PA-dPEG24** | **IALILEPICCQERAA-dPEG24** | **Yes** | **2.99** |
| PA-dPEG20 | IALILEPICCQERAA-dPEG20 | Yes | 11.41 |
| PA-dPEG16 | IALILEPICCQERAA-dPEG16 | Yes | 12.04 |
| PA-dPEG12 | IALILEPICCQERAA-dPEG12 | Yes | 11.41 |
| PA-dPEG08 | IALILEPICCQERAA-dPEG08 | Yes | 34.66 |
| PA-dPEG06 | IALILEPICCQERAA-dPEG06 | No | 44.82 |
| PA-dPEG04 | IALILEPICCQERAA-dPEG04 | Yes | 12.36 |
| PA-dPEG03 | IALILEPICCQERAA-dPEG03 | Yes | 12.57 |
| PA-dPEG02 | IALILEPICCQERAA-dPEG02 | Yes | 11.62 |

The final concentration of peptide in factor B-depleted serum was 0.77mM.

^1^Peptides not soluble in water were resuspended in DMSO.

^2^In the hemolytic assay, soluble peptides are standardized to water and insoluble peptides standardized to DMSO.
