# Supplementary material for: Peptide Inhibitor of Complement C1 (PIC1) Rapidly Inhibits Complement Activation after Intravascular Injection in Rats
Source: PLoS One. 2015 Jul 21;10(7):e0132446. doi: 10.1371/journal.pone.0132446 (PMC4511006; doi:10.1371/journal.pone.0132446)
Supplement: S2 Table — (DOCX) [file pone.0132446.s002.docx]

**S2 Table. PA-dPEG24 toxicology evaluation at 48 hr (ranges).**

|  | **PA-dPEG24 (n=3)** | **Saline (n=3)** |
| --- | --- | --- |
| WBC | 6.4 – 7.7 | 8.5 – 9.2 |
| RBC | 5.7 – 5.8 | 5.6 – 5.9 |
| platelets | 1036 – 1289 | 1120 – 1332 |
| AST | 100 – 108 | 63 –123 |
| ALT | 41 – 50 | 21 – 48 |
| Alk phos | 111 – 133 | 101 – 123 |
| Bilirubin | 0.1 | 0.1 |
| GGT | 1 | 1 – 2 |
| BUN | 10 – 15 | 11 – 20 |
| Creatinine | 0.3 | 0.3 |
| Lipase | 18 – 29 | 23 – 165 |
