# Supplementary figures and images for: Peptide Inhibitor of Complement C1 (PIC1) Rapidly Inhibits Complement Activation after Intravascular Injection in Rats
Source: PLoS One. 2015 Jul 21;10(7):e0132446. doi: 10.1371/journal.pone.0132446 (PMC4511006; doi:10.1371/journal.pone.0132446)

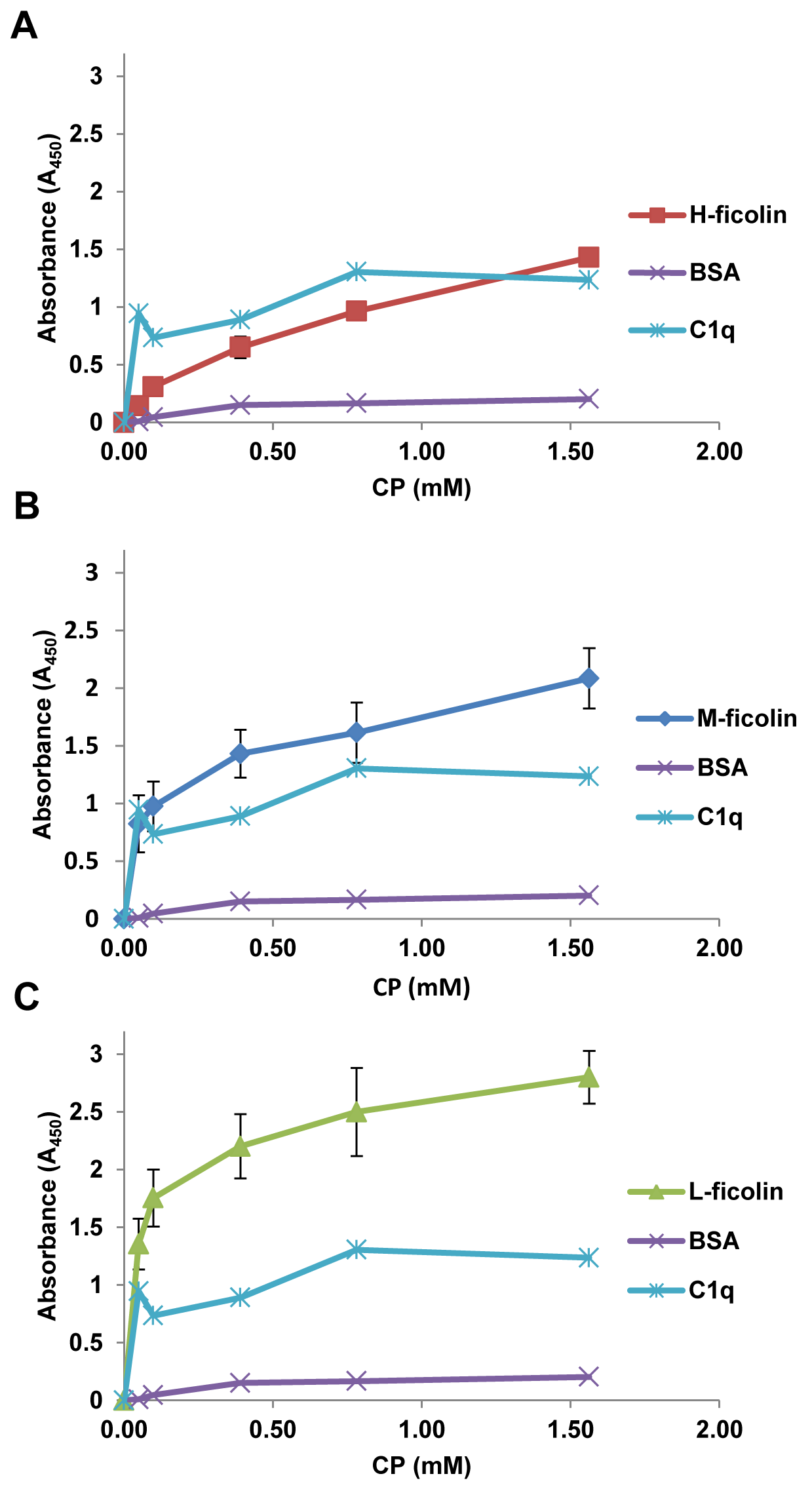

Supplement: S1 Fig — The various ficolins indicated in the figure were adsorbed to the microtiter plate and incubated with CP followed by antibody to CP. BSA was used as a negative control for binding and C1q as a positive control for binding. Ficolin data represent the means of three independent experiments with error bars denote SEM. BSA and C1q values were from two independent experiments. (TIF) [file pone.0132446.s003.tif]

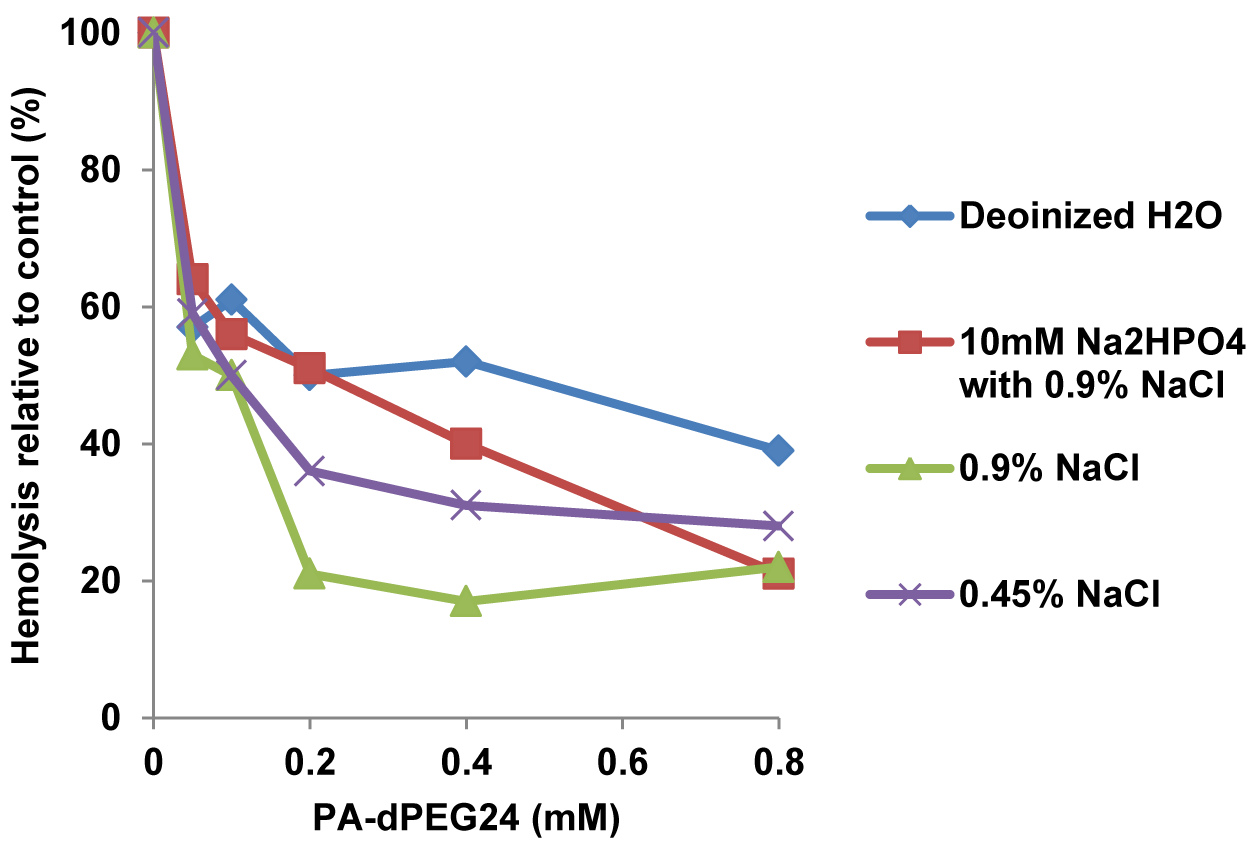

Supplement: S2 Fig — Hemolytic assays using Wistar rat serum were performed with PA-dPEG24 dissolved in 0.9% NaCl, 0.45% NaCl, 10 mM Na2HPO4 with 0.9% saline or water. The sera were incubated with peptide and then added to human AB erythrocytes. (TIF) [file pone.0132446.s004.tif]
